# Outcomes of Soft-Tissue Reconstruction Around the Elbow Using a Lateral Intercostal Artery Perforator-Based Pedicled Flap

**DOI:** 10.7759/cureus.117371

**Published:** 2026-10-02

**Authors:** Faruk Hossain, Md Razaul Karim, Fatema Mirza, Md Abu Nahiyan, Kamruzzaman Akash, Md Ashraful Islam Irfan, Rakibul Islam, Tahmina Satter

**Affiliations:** 1 Department of Burn and Plastic Surgery, Dhaka Community Medical College, Dhaka, BGD; 2 Department of Plastic Surgery, National Institute of Traumatology and Orthopaedic Rehabilitation, Dhaka, BGD; 3 Department of Burn and Plastic Surgery, Dhaka Medical College and Hospital, Dhaka, BGD; 4 Department of Plastic Surgery, Mirza Plastic Surgery Center, Dhaka, BGD; 5 Department of Medicine, Dhaka Community Medical College Hospital, Dhaka, BGD; 6 Medical Strategy, Noora Health, Dhaka, BGD

**Keywords:** cubital fossa, elbow reconstruction, lateral intercostal artery perforator, licap flap, pedicled flap, reconstructive surgery, soft-tissue defect

## Abstract

Background

Soft-tissue defects around the elbow present considerable reconstructive challenges, as effective coverage must be achieved without compromising joint mobility. This study assessed the reconstructive and early functional outcomes of lateral intercostal artery perforator (LICAP)-based pedicled flaps for soft-tissue reconstruction around the elbow.

Methods

This prospective observational study included 30 consecutively enrolled patients who underwent LICAP-based pedicled flap reconstruction at the Department of Plastic Surgery, Dhaka Medical College Hospital, Dhaka, Bangladesh, between May 2023 and October 2024. Evaluated outcomes comprised flap survival, flap-related complications, donor-site morbidity, elbow range of motion (ROM), and a predefined composite reconstructive outcome. Elbow ROM was measured with a goniometer at the final follow-up, approximately 30 days after flap division.

Results

The mean patient age was 27.4 ± 10.97 years, and 26 patients were male. Trauma was the cause of 18 defects. Complete flap survival was observed in 27 patients, whereas marginal and partial flap loss occurred in two and one patient, respectively; no patient experienced significant flap loss. Recipient-site wound dehiscence developed in two patients, and donor-site wound dehiscence occurred in three. Primary closure of the donor site was achieved in 29 patients. At the final follow-up, 26 patients demonstrated full elbow ROM, while four had a residual restriction of 10°-15°. Composite outcomes were classified as good in 22 patients and satisfactory in eight; no poor outcomes were recorded.

Conclusions

In appropriately selected patients, LICAP-based pedicled flap reconstruction provided favorable early reconstructive and functional outcomes for soft-tissue defects around the elbow. Comparative studies involving larger patient populations and extended follow-up are warranted.

## Introduction

Soft-tissue defects in and around the cubital fossa may result from trauma, burns, post-burn contracture release, infection, or tumor excision [1]. Reconstruction of defects in this region is particularly challenging because of the complex anatomy and functional importance of the elbow. Such defects may expose bone, tendons, joints, neurovascular structures, or implanted hardware, necessitating durable soft-tissue coverage [2]. An ideal reconstruction should provide stable, well-vascularized, and sufficiently pliable tissue while minimizing secondary contracture and permitting restoration of elbow mobility [3].

Post-burn contracture represents an additional reconstructive challenge. Following adequate scar release, skin grafting may be appropriate for superficial defects when vital structures remain adequately covered; however, larger or deeper defects, particularly those with exposed critical structures, may require flap coverage [4,5]. Flap reconstruction provides vascularized tissue and may offer durable coverage across a mobile joint where recurrent contracture and functional limitation are important concerns [3-5].

Several local and regional flaps have been described for reconstruction around the elbow, including lateral arm, radial artery, posterior interosseous artery, transposition, and local muscle flaps [2,6]. The reverse-flow lateral arm flap is also a useful regional option for elbow defects, with successful clinical application reported in both pediatric elbow defects and post-burn antecubital contractures [7,8]. However, the choice of a local or regional flap depends on the size and location of the defect, the condition of the surrounding tissues, previous trauma or burns, vascular availability, and donor-site considerations [5-8]. Consequently, extensive local tissue injury or scarring may limit the availability of suitable tissue adjacent to the defect.

Distant pedicled flaps remain valuable when local or regional reconstructive options are unsuitable. A systematic review of distant pedicled flaps for elbow coverage identified several abdominal and thoracoabdominal options and highlighted their continuing role in selected defects that are not amenable to regional or free-flap reconstruction [9]. Lateral intercostal artery perforator (LICAP)-based flaps represent another option for transferring vascularized fasciocutaneous tissue from the lateral or posterolateral thoracic region. Importantly, Acartürk described a LICAP-based reverse thoracic flap for reconstruction of an antecubital defect, demonstrating the feasibility of this perforator-based pedicled approach for coverage of the antecubital region [10].

Despite its potential utility, published evidence specifically addressing LICAP-based pedicled flap reconstruction for soft-tissue defects in and around the cubital fossa remains limited. Therefore, this study aimed to evaluate the reconstructive and functional outcomes of LICAP-based pedicled flap reconstruction for soft-tissue defects in and around the cubital fossa, with particular emphasis on flap survival, postoperative complications, donor-site morbidity, elbow range of motion (ROM), and the predefined composite reconstructive outcome.

## Materials and methods

Study design and setting

This prospective observational study was conducted in the Department of Plastic Surgery at Dhaka Medical College Hospital (DMCH) from May 2023 to October 2024. It assessed the reconstructive and functional outcomes of LICAP-based pedicled flaps used to repair soft-tissue defects around the elbow joint.

Study population and sampling

Patients with elbow-area soft-tissue defects were assessed for eligibility. These included defects caused by trauma, burns, post-burn wounds, scar contracture releases, tumor removal, infection, or other causes requiring soft-tissue coverage around the elbow. Patients with defects that exposed or involved critical structures such as vessels, nerves, tendons, or bone and those with limited flap options were considered suitable candidates for LICAP-based reconstruction. During the study, 38 patients were evaluated; eight were excluded based on the criteria. The remaining 30 eligible patients underwent LICAP reconstruction and were included in the final analysis (Figure 1).

**Figure 1:**
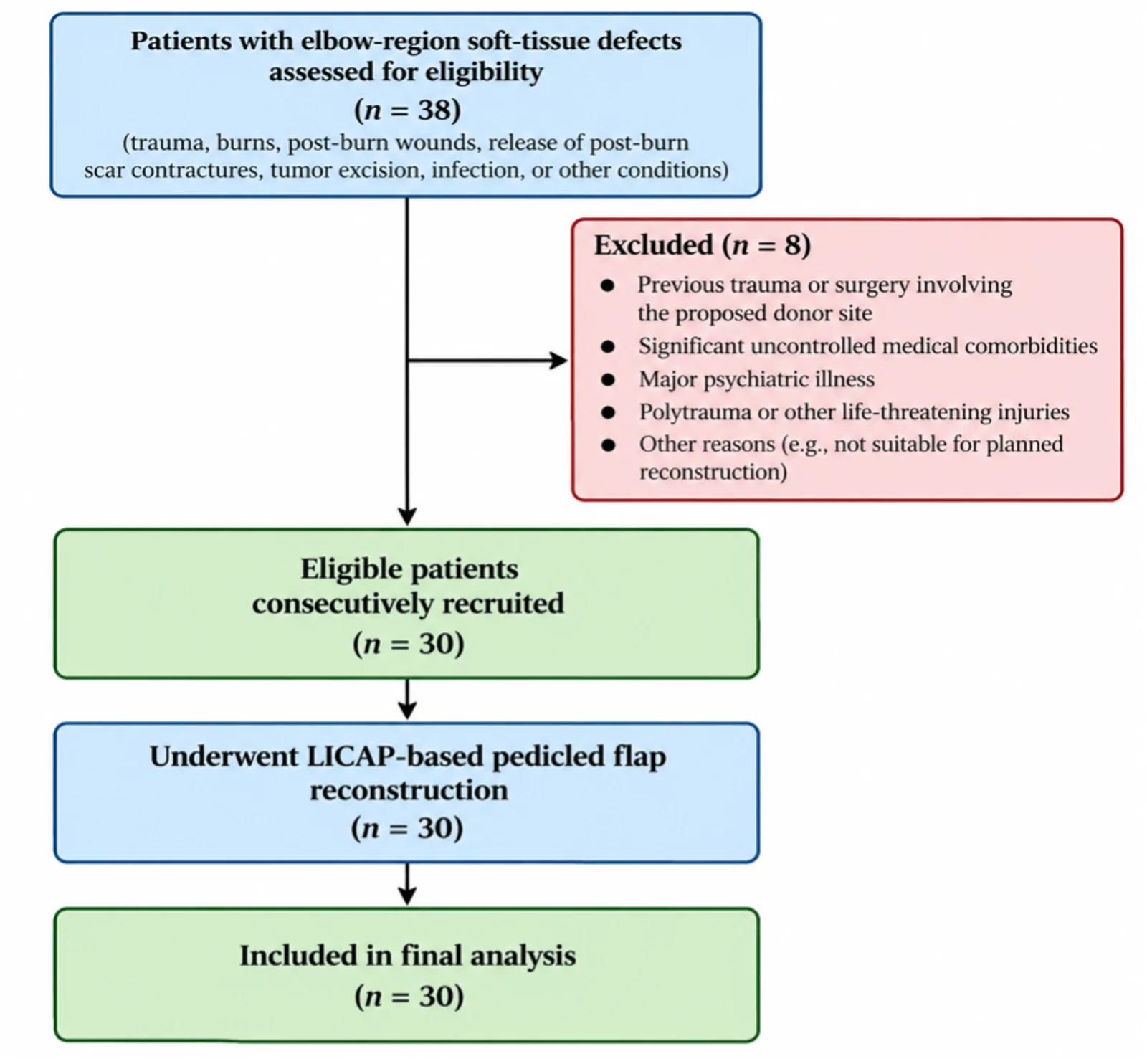
Cohort Flowchart Illustrating Patient Assessment, Eligibility, Recruitment, and Inclusion in the Final Analysis LICAP, lateral intercostal artery perforator.

Sample size

The sample size was calculated using the finite population single-proportion formula, $n = \frac{Z^2pqN}{d^2(N-1) + Z^2pq}$, where p represents the assumed proportion, q = 1-p, and d represents the desired absolute precision. In the absence of sufficiently robust prior data specific to LICAP-based pedicled flap reconstruction around the elbow, we used an assumed proportion (p) of 25% as a planning parameter. This proportion was not intended to represent a specific previously reported clinical outcome for LICAP-based elbow reconstruction. Based on an average institutional case volume of approximately three eligible patients per month and an 18-month study period, approximately 54 eligible patients were anticipated. Using a 95% confidence level (Z = 1.96) and an absolute precision (d) of 10%, the calculated sample size was 31.11, approximately 31 patients. Ultimately, 30 eligible patients were consecutively enrolled and included in the final analysis.

Eligibility criteria

Patients were eligible if they had a soft-tissue defect in or around the cubital fossa with exposure of critical structures, including vessels, nerves, tendons, or bone, resulting from trauma, scar-contracture release, burn, tumor excision, infection, or other causes and requiring flap coverage. Patients were also eligible when the defect had limited suitable local flap options for achieving adequate soft-tissue coverage. The suitability of the LICAP-based pedicled flap was determined clinically by the treating reconstructive surgical team based on the characteristics of the defect and the availability of local flap options.

Patients were excluded if they had trauma or previous surgery at the donor site, uncontrolled medical conditions, major psychiatric illnesses, polytrauma, or other severe injuries.

Preoperative assessment and flap planning

All patients underwent a medical history review, clinical examination, and relevant investigations to characterize the defect and assess surgical fitness. General health, nutritional status, and wound condition were optimized before definitive reconstruction. The etiology and laterality of each defect were recorded.

After wound debridement or release and excision of scar contracture, the maximum longitudinal and perpendicular dimensions of the resulting defect were measured in centimeters using a measuring scale. Defect area was estimated by multiplying these dimensions. Flap dimensions were recorded using the same method. A representative post-traumatic defect is shown in Figure 2A. A handheld vascular Doppler was used to identify and mark the LICAP in the midaxillary region. The flap was designed over the posterolateral thoracic region according to the dimensions of the recipient defect and the location of the Doppler-identified perforator (Figure 2B).

**Figure 2:**
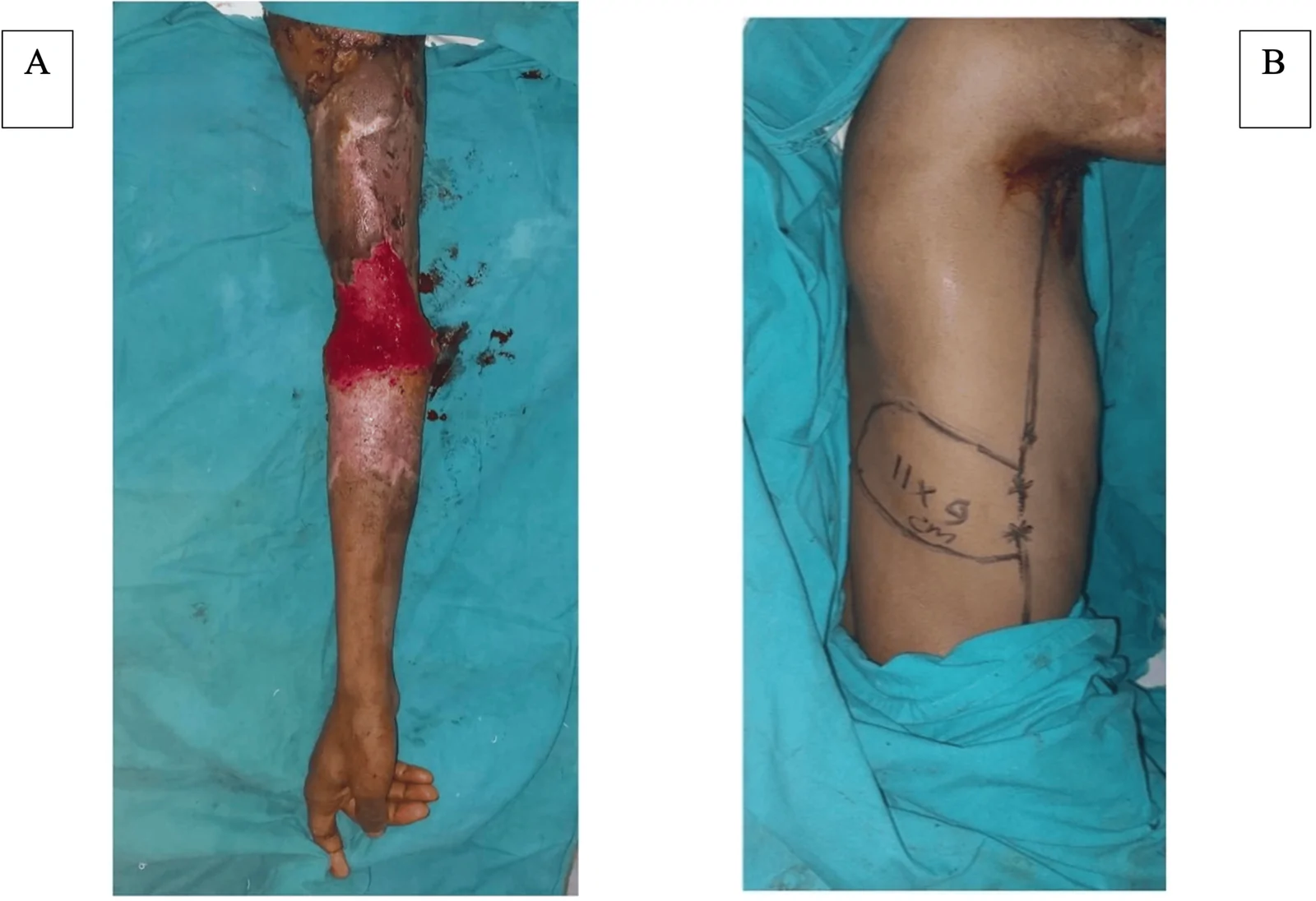
Defect Assessment and Planning of the LICAP-Based Pedicled Flap (A) Soft-tissue defect around the elbow joint following trauma. (B) Design of the LICAP-based pedicled flap over the posterolateral thoracic region according to the dimensions of the recipient defect and the location of the Doppler-identified perforator. Source: Authors’ own clinical photographs, obtained during the present study with consent for publication. LICAP, lateral intercostal artery perforator

Operative technique

All procedures were conducted under general anesthesia. Initial wound debridement or release and excision of the scar contracture were performed with the patient in the supine position to prepare an adequate recipient bed and establish the definitive dimensions of the defect. The patient was then repositioned laterally, with the affected upper limb freely draped to facilitate flap elevation and transfer.

The LICAP was identified at the midaxillary line using a handheld Doppler. The flap was designed over the lateral thoracic region based on the dimensions of the resulting defect. To compensate for the reduction in effective flap length during transfer and inset, the flap length was planned to be approximately 2 cm greater than the defect length. Its width was designed to be approximately 1 cm greater than the defect width to permit tension-free coverage. The posterior border was positioned close to, but without crossing, the posterior midline.

The flap was elevated transversely and parallel to the ribs. The posterior incision was made first, followed by dissection from posterior to anterior under loupe magnification. Flap elevation was performed in the subfascial plane to preserve the vascular supply. At the anterior border of the latissimus dorsi muscle, the posterior cutaneous branch of the perforator was identified and preserved. After flap elevation, distal flap perfusion was assessed clinically based on the appearance and bleeding of the distal flap margin. The handheld Doppler was not used for postoperative flap monitoring.

The donor site was assessed intraoperatively for primary closure after flap elevation. Primary closure was performed when the donor-site edges could be approximated without excessive tension and was generally achievable for flap widths of up to approximately 9 cm. When primary closure could not be performed without excessive tension, the remaining donor-site defect was covered with a split-thickness skin graft (STSG).

The affected upper limb was subsequently adducted toward the trunk in a comfortable position. The pedicled flap was transferred to the cubital fossa defect and inset without undue tension (Figure 3A). Particular care was taken to prevent compression, kinking, or excessive tension of the vascular pedicle. The pedicle was loosely secured to cover the exposed raw surfaces, and the upper limb was maintained in adduction throughout the period of pedicle attachment. The postoperative appearance following flap division is shown in Figure 3B. Flap division was performed on postoperative day 21 after clinical confirmation of satisfactory flap viability. Following division, the flap was definitively inset and sutured to the recipient site (Figure 3C).

**Figure 3:**
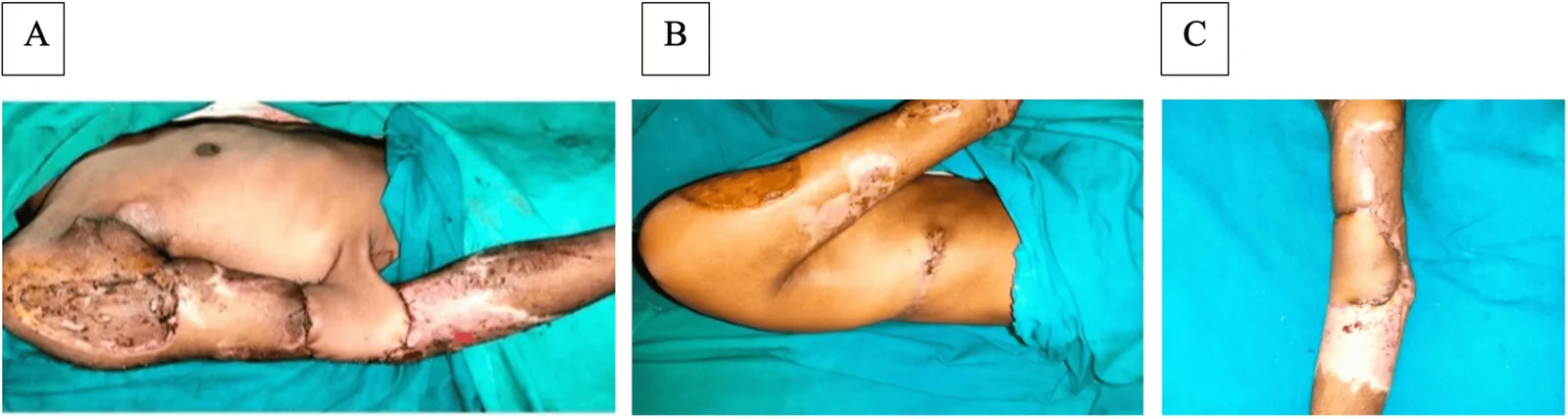
Transfer, Temporary Inset, and Subsequent Division of the LICAP-Based Pedicled Flap (A) Transfer and inset of the pedicled flap into the elbow joint defect with the upper limb maintained in adduction to the trunk. (B) Postoperative appearance of the transferred flap during the period of pedicle attachment. (C) Recipient-site appearance following flap division and definitive inset. Source: Authors’ own clinical photographs, obtained during the present study with consent for publication. LICAP, lateral intercostal artery perforator

Postoperative management and follow-Up

Intravenous antibiotics were administered perioperatively and continued during the early postoperative period for seven days, according to the clinical condition of the wound. Following that, oral antibiotics were continued for another 7-10 days. Wound swab for culture and antimicrobial susceptibility testing was done when postoperative wound infection was clinically suspected, particularly in the presence of purulent discharge, increasing erythema, or other clinical evidence of infection. Antibiotic therapy was subsequently continued or modified according to the culture and susceptibility results when available. Routine wound cultures were not performed in clinically uninfected wounds.

Patients were clinically evaluated on postoperative days 1, 5, and 21, with subsequent follow-up at approximately postoperative days 35 and 50. Assessments included flap viability, recipient-site healing (Figure 4B), flap-related complications, and donor-site morbidity (Figures 4A, 4C). Flap division was performed on postoperative day 21. Sutures were removed approximately 10 days after flap division, and elbow physiotherapy was initiated at the same time.

**Figure 4:**
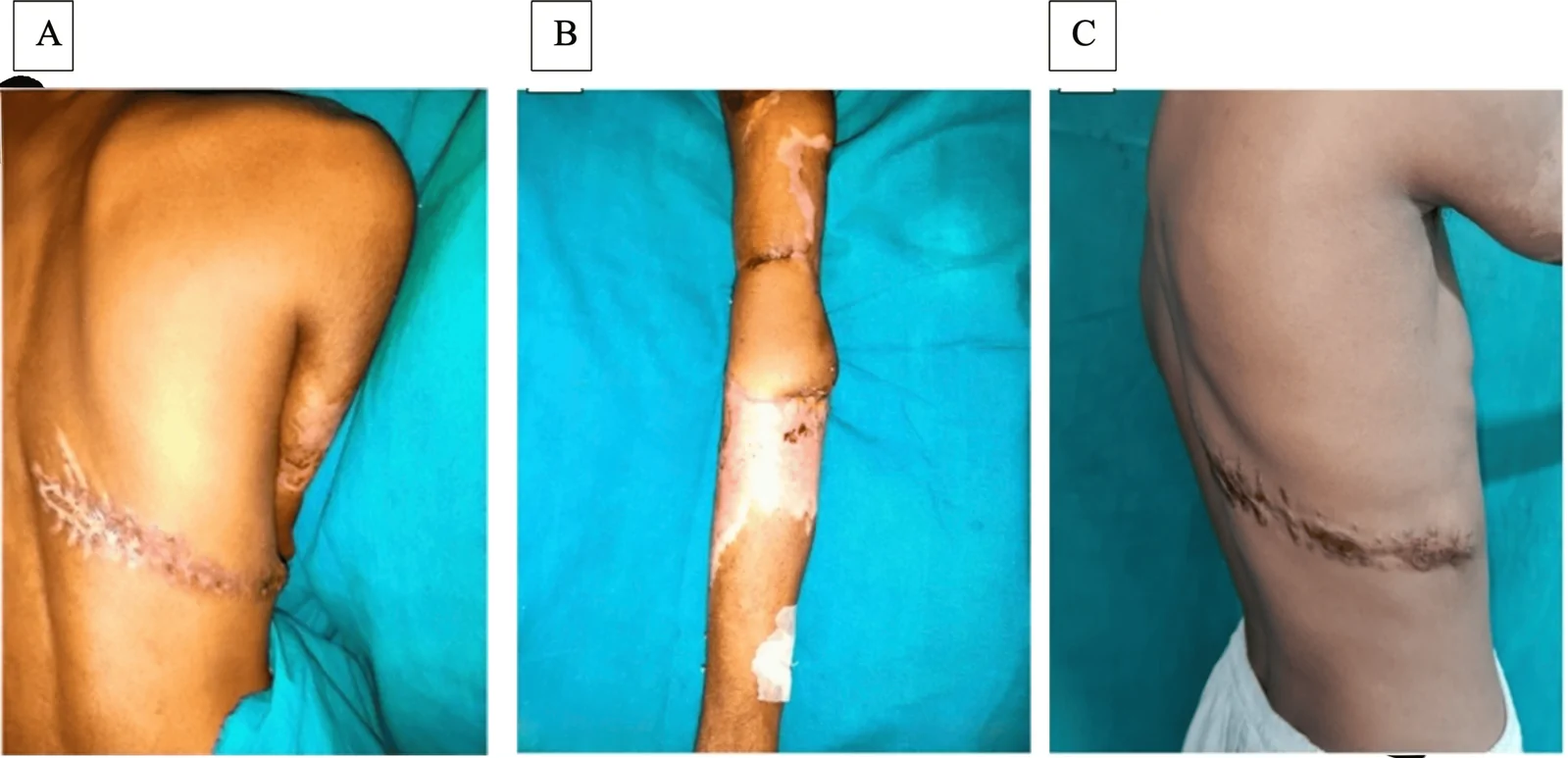
Postoperative Recipient- and Donor-Site Outcomes Following LICAP-Based Pedicled Flap Reconstruction (A, C) Follow-up appearance of the lateral thoracic donor site demonstrating donor-site healing. (B) Follow-up appearance of the reconstructed elbow joint demonstrating flap survival and recipient-site healing. Source: Authors’ own clinical photographs, obtained during the present study with consent for publication. LICAP, lateral intercostal artery perforator

Elbow ROM was measured using a goniometer at the final follow-up, approximately 30 days after flap division.

Outcome measures

The principal outcome measures were flap survival, flap-related complications, donor-site morbidity, elbow ROM, and composite reconstructive outcome.

Flap survival

Flap viability was assessed clinically throughout the postoperative follow-up period. Flap survival was classified according to the extent of flap loss. Complete flap survival was defined as the absence of flap loss. Marginal flap loss was defined as <10% flap loss, partial flap loss as 10%-30% flap loss, and significant flap loss as >30% flap loss, based on a previously reported flap-survival classification [11].

Flap-related complications

Flap-related complications included venous congestion and recipient-site wound dehiscence. Wound dehiscence was defined as partial or complete disruption of one or more layers of the operative wound.

Donor-site outcomes

Donor-site outcomes included the method of donor-site closure and postoperative donor-site complications. Donor-site closure was categorized as primary closure or STSG. Recorded donor-site complications included wound dehiscence and graft loss.

Functional outcome

Elbow ROM was assessed using a goniometer at the final study follow-up, approximately 30 days after flap division (approximately 50-51 days postoperatively). As joint mobilization was initiated approximately 10 days after flap division, this assessment represented an early postoperative evaluation of elbow mobility rather than definitive long-term functional recovery. Functional outcome based on elbow ROM was categorized as full ROM, restricted movement, or complete absence of elbow movement. In patients with restricted movement, the residual limitation was quantified in degrees. Validated functional or patient-reported outcome measures, such as the Mayo Elbow Performance Score (MEPS) or Disabilities of the Arm, Shoulder and Hand (DASH) questionnaire, were not used in this study.

Composite reconstructive outcome

The overall reconstructive outcome was assessed using predefined criteria incorporating flap survival, flap-related complications, donor-site morbidity, and elbow function, consistent with multidimensional assessment of soft-tissue reconstruction outcomes [12,13].

A good outcome was defined as complete flap survival, absence of flap-related and donor-site complications, and full elbow ROM. A satisfactory outcome was defined as marginal or partial flap loss, conservatively managed flap-related or donor-site complications, and/or residual restriction of elbow movement. A poor outcome was defined as significant flap loss, complications requiring surgical intervention, and/or complete loss of elbow movement.

These composite criteria were predefined by the investigators for the present study and were not externally validated.

Data collection

Demographic, clinical, operative, and postoperative data were gathered prospectively using a semi-structured form created by the study investigators specifically for this research. The form was designed based on the study objectives and included variables such as patient characteristics, defect etiology and features, surgical details, flap survival, complications related to the flap and donor site, elbow ROM, and overall reconstructive outcomes. The full data collection form is available in the Appendix section.

Patients were evaluated during postoperative follow-up to check flap viability, healing at the recipient site, potential flap complications, and donor-site morbidity. About 30 days after flap division, at the final follow-up, elbow ROM was measured with a goniometer and classified as full, restricted, or absent. For those with limited movement, the exact degree of restriction was noted in degrees. Data were reviewed for completeness and consistency before statistical analysis.

Statistical analysis

Data entry, verification, processing, and analysis were performed using IBM SPSS Statistics for Windows, Version 26.0 (Released 2018; IBM Corp., Armonk, NY, USA). Continuous variables were summarized using the mean, standard deviation, and range, whereas categorical variables were presented as frequencies and percentages. Given the single-arm prospective observational design and the descriptive objective of the study, the analysis was limited primarily to descriptive statistics. Comparative and multivariable inferential analyses were not performed. The analysis focused on flap survival, postoperative complications, donor-site morbidity, elbow function, and the predefined composite reconstructive outcome. Percentages were rounded to one decimal place.

## Results

Baseline demographic and clinical characteristics

A total of 30 patients underwent reconstruction of soft-tissue defects around the elbow joint using a LICAP-based pedicled flap. The mean age was 27.4 ± 10.97 years, and 26 (86.7%) patients were male. The 21-30-year age group was the largest, comprising 10 (33.3%) patients. Trauma appeared to be the predominant cause of the defect, accounting for 18 (60.0%) cases, followed by post-burn scar contracture release in 11 (36.7%) and a post-burn wound in one (3.3%). Eighteen (60.0%) defects involved the right side, while 12 (40.0%) involved the left side (Table 1).

**Table 1:** Baseline Demographic and Clinical Characteristics of the Study Population (N = 30) Data are presented as frequencies (n) and percentages (%) unless otherwise indicated. SD, standard deviation

Characteristic	n	%
Age group, years		
≤20	7	23.3
21-30	10	33.3
31-40	8	26.7
41-50	5	16.7
Age, mean ± SD, years	27.4 ± 10.97	-
Sex		
Male	26	86.7
Female	4	13.3
Comorbidities		
Hypertension	2	6.7
Diabetes mellitus	1	3.3
Cause of defect		
Trauma	18	60.0
Post-burn scar contracture release	11	36.7
Post-burn wound	1	3.3
Side of defect		
Right	18	60.0
Left	12	40.0

Defect, flap, and donor-site characteristics

The mean defect area was 57.3 ± 16.48 cm² (range, 35-84 cm²), while the mean flap area was 80.23 ± 18.96 cm² (range, 54-112 cm²). Primary donor-site closure was achieved in 29 (96.7%) patients. Only one patient (3.3%) required STSG for donor-site coverage (Table 2).

**Table 2:** Defect, Flap, and Donor-Site Characteristics (N = 30) Continuous variables are presented as mean ± standard deviation (SD) and range. Donor-site closure is presented as frequency (n) and percentage (%). STSG, split-thickness skin graft

Characteristic	Value
Defect area, mean ± SD, cm²	57.3 ± 16.48
Defect area, range, cm²	35-84
Flap area, mean ± SD, cm²	80.23 ± 18.96
Flap area, range, cm²	54-112
Primary donor-site closure, n (%)	29 (96.7)
STSG, n (%)	1 (3.3)

Flap survival and postoperative complications

Each patient was assigned a single composite outcome category according to the predefined criteria, irrespective of the number of individual outcome domains affected. Complete flap survival was achieved in 27 (90.0%) patients. Marginal flap loss occurred in two (6.7%) patients, whereas partial flap loss occurred in one (3.3%). No patient developed significant flap loss.

Recipient-site wound dehiscence occurred in two (6.7%) patients, whereas no venous congestion was recorded. Overall, the findings suggest that 28 (93.3%) patients had no recorded flap-related complication. Donor-site wound dehiscence was observed in three (10.0%) patients, whereas graft loss was not recorded. The findings suggest that 27 (90.0%) patients had no recorded donor-site complication (Table 3).

**Table 3:** Flap Survival and Postoperative Complications (N = 30) Marginal flap loss was defined as <10% distal flap loss, partial flap loss as 10%-30% distal flap loss, and significant flap loss as >30%. Flap survival, flap-related complications, and donor-site complications represent separate outcome domains.

Outcome	n	%
Flap survival		
Complete flap survival	27	90.0
Marginal flap loss	2	6.7
Partial flap loss	1	3.3
Significant flap loss	0	0.0
Flap-related complications		
Wound dehiscence	2	6.7
Venous congestion	0	0.0
No flap-related complication	28	93.3
Donor-site complications		
Wound dehiscence	3	10.0
Graft loss	0	0.0
No donor-site complication	27	90.0

Functional and composite reconstructive outcomes

At the final study assessment, 26 patients (86.7%) had full elbow ROM, while four patients (13.3%) had restricted movement with a residual limitation of 10°-15°. No patient had complete loss of elbow movement.

Based on the predefined criteria incorporating flap survival, flap-related complications, donor-site morbidity, and elbow function, 22 (73.3%) patients achieved a good composite reconstructive outcome, while eight (26.7%) achieved a satisfactory outcome. The reasons for classification as satisfactory included marginal or partial flap loss, conservatively managed postoperative complications, and/or residual restriction of elbow movement. No patient met the criteria for a poor composite reconstructive outcome (Table 4).

**Table 4:** Functional and Composite Reconstructive Outcomes (N = 30) Elbow range of motion was assessed using a goniometer at the final follow-up, approximately 30 days after flap division. The composite reconstructive outcome incorporated flap survival, flap-related complications, donor-site morbidity, and elbow function according to the predefined study criteria.

Outcome	n	%
Elbow range of motion at final follow-up		
Full range of motion	26	86.7
Restricted by 10°-15°	4	13.3
No movement	0	0.0
Composite reconstructive outcome		
Good	22	73.3
Satisfactory	8	26.7
Poor	0	0.0

## Discussion

This prospective observational study evaluated the reconstructive and early functional outcomes of LICAP-based pedicled flaps for soft-tissue reconstruction around the elbow. The principal findings were 90.0% complete flap survival, absence of significant flap loss, limited recipient- and donor-site morbidity, primary donor-site closure in 96.7% of patients, and full elbow ROM in 86.7%. In addition, 73.3% of patients achieved a good composite reconstructive outcome, and none had a poor outcome. Taken together, these findings suggest that the LICAP-based pedicled flap may constitute a feasible option for appropriately selected soft-tissue defects around the elbow.

The reconstruction of soft-tissue defects around the elbow presents considerable challenges in that durable vascularized coverage must be achieved while preserving mobility across a highly functional joint. Trauma, burns, and release of post-burn contractures may produce defects with exposure of critical structures, while adjacent tissues may be scarred or unsuitable for local reconstruction. Several local, regional, distant pedicled, and free-flap options have therefore been described [2,5,6]. Contemporary reconstructive principles emphasize individualized flap selection according to defect characteristics, condition of the surrounding tissues, vascular availability, donor-site morbidity, and surgical expertise [14]. The LICAP-based pedicled flap is particularly relevant when local tissue is compromised because it provides vascularized fasciocutaneous tissue from outside the immediate zone of injury without requiring microvascular anastomosis.

Trauma was the predominant indication in the present series, followed by defects resulting from release of post-burn scar contracture. The defects ranged from 35 to 84 cm², and the flap was designed slightly larger than the recipient defect to facilitate tension-free transfer and inset. Previous peri-elbow reconstruction using a lateral thoracic flap included defects ranging approximately from 5 × 4 cm to 12 × 10 cm [15], while LICAP-based pedicled reconstruction has likewise been applied, with reported success, to antecubital burn contractures [16]. Together with previous reports of regional flaps and LICAP-based antecubital reconstruction [7,8,10], these findings support the versatility of lateral thoracic tissue for coverage of moderate-sized defects when adjacent reconstructive options are limited.

Complete flap survival was achieved in 27 (90.0%) patients. The remaining three patients developed marginal or partial flap loss, and no significant flap loss occurred. Previous peri-elbow lateral thoracic flap reconstruction has similarly demonstrated generally favorable flap survival, although partial flap loss requiring secondary management has been reported [15]. Favorable survival has also been described with LICAP-based pedicled reconstruction of antecubital defects [10,16]. In the present study, recipient-site wound dehiscence occurred in two patients, while marginal or partial flap loss occurred in three patients. These complications occurred in different patients, with no individual patient experiencing both flap loss and recipient-site wound dehiscence. No venous congestion was recorded. Preoperative Doppler localization, preservation of the perforator during subfascial elevation, and avoidance of excessive tension during transfer were integral components of the operative technique. However, the present observational design does not permit attribution of the favorable outcomes to any individual technical factor.

Donor-site morbidity was limited. Primary closure was possible in 29 (96.7%) patients, with only one patient requiring STSG. Donor-site wound dehiscence occurred in three patients and was managed conservatively, while no graft loss was observed. Previous LICAP-based reconstruction has also demonstrated that the donor site can often be closed primarily [10,16]. This may represent a practical advantage of the lateral thoracic donor region, particularly when flap width permits direct closure. The relatively pliable fasciocutaneous tissue may also be advantageous for coverage around a mobile joint [10,16]. Nevertheless, although donor-site healing was favorable in this series, aesthetic outcomes, scar quality, and patient satisfaction were not formally evaluated.

Functional recovery is an important endpoint in elbow reconstruction because flap survival alone does not necessarily indicate a successful outcome. At final assessment, 26 patients had full elbow ROM, while four had residual restrictions of 10°-15°; no patient had complete loss of movement. Favorable functional recovery following lateral thoracic flap reconstruction of peri-elbow defects has also been reported [15]. More broadly, stable soft-tissue coverage followed by appropriate rehabilitation may help preserve or restore elbow mobility after burn contracture release and upper-extremity reconstruction [3,5]. These findings are encouraging but should be interpreted as early postoperative outcomes, given that the assessment was performed approximately 30 days after flap division. Future prospective studies should combine goniometric assessment with validated functional and patient-reported outcome measures to provide a more comprehensive evaluation of recovery.

The predefined composite outcome incorporated flap survival, flap-related complications, donor-site morbidity, and elbow function, thereby providing a broader assessment of reconstructive success than flap survival alone. A good composite outcome was achieved in 22 (73.3%) patients, while eight (26.7%) had a satisfactory outcome and none had a poor outcome. The lower proportion of good composite outcomes compared with complete flap survival suggests that technical flap viability alone may overestimate overall reconstructive success when functional and donor-site outcomes are not considered. However, because this composite classification was developed for the present study and has not been externally validated, it should be regarded as a descriptive clinical measure rather than a standardized outcome instrument.

The LICAP-based pedicled flap may offer several practical advantages. Compared with abdominal-based flaps, it has been described as relatively thin, which may facilitate contour matching and reduce excessive bulk, while the donor-site scar may be positioned in a relatively inconspicuous location [16,17]. However, these aesthetic and contour-related outcomes were not formally evaluated in the present study. Furthermore, the donor-site scar is located posteriorly and can be concealed in a relatively inconspicuous area, making it cosmetically more acceptable in appropriately selected patients. It may provide vascularized tissue from outside the traumatized or scarred region, may not require microvascular anastomosis, and appears to have permitted primary donor-site closure in nearly all patients in this series. Previous descriptions of LICAP-based antecubital reconstruction similarly highlight the utility of lateral thoracic tissue for this purpose [10,18]. These advantages must be weighed against important disadvantages, including the requirement for a staged procedure, temporary attachment of the upper limb to the trunk, and delayed mobilization until flap division. Therefore, the LICAP-based pedicled flap should be regarded as one option within the reconstructive armamentarium rather than a replacement for established local, regional, or free-flap techniques [14].

Several limitations should be considered when interpreting these findings. The relatively short follow-up limited assessment of long-term flap durability, recurrent contracture, scar maturation, and sustained elbow function. Elbow ROM was assessed approximately 30 days after flap division and therefore represents an early postoperative assessment rather than definitive long-term functional recovery. Longer follow-up would be required to evaluate sustained ROM and recurrent contracture. Selection bias is possible because only patients considered clinically suitable for LICAP-based pedicled flap reconstruction were included, which may limit generalizability to patients managed with other reconstructive approaches. In addition, validated functional and patient-reported outcome measures were not used, and aesthetic outcomes and patient satisfaction regarding the recipient and donor sites were not formally assessed. The small sample size and single-center setting may further limit generalizability, while the absence of a comparison group precludes direct comparison with other established reconstructive options.

## Conclusions

The LICAP-based pedicled flap was associated with favorable early reconstructive outcomes in this selected patient series, with high flap survival, manageable recipient- and donor-site complications, and favorable early elbow range-of-motion findings. Most patients achieved primary donor-site closure, and the majority had full elbow motion at the final early postoperative assessment. These findings suggest that LICAP-based pedicled flaps may be a viable reconstructive option for carefully selected patients with peri-elbow soft-tissue defects, particularly when local tissue is insufficient or unsuitable. However, given the small sample size, single-centre setting, observational design, and absence of a comparison group, these findings should not be interpreted as evidence of comparative effectiveness. Larger comparative studies with longer follow-up and validated functional and patient-reported outcome measures are warranted to further evaluate long-term functional and aesthetic outcomes and define the role of LICAP-based reconstruction relative to other reconstructive options.

## Appendices

Appendix

**Table 5:** Data Collection Sheet Used in the Study Before starting the interview, the researcher explained that the participant would first be asked some background questions, followed by questions about the elbow soft-tissue defect, surgical treatment, postoperative recovery, and functional outcome. The participant was informed that all information provided would be kept confidential and used solely for research purposes.

Section	No.	Variable/Question	Response/Options
A. Administrative Information	1	Serial No.	__________________
	2	Date of assessment	__________________
	3	Patient Registration No.	__________________
B. Demographic and Clinical Characteristics	4	Age	______ years
	5	Sex	☐ Male ☐ Female
	6	Cause of defect	☐ Trauma ☐ Burn ☐ Postoperative ☐ Post-infective ☐ Tumor excision ☐ Other: __________
	7	Side of defect	☐ Right ☐ Left
	8	Site of defect	☐ Cubital fossa ☐ Flexor surface ☐ Extensor surface
	9	Duration of wound	______ months ______ days
	10	Comorbidities	☐ None ☐ Diabetes mellitus ☐ Hypertension ☐ Vascular disease ☐ Other: __________
	11	Exposed structure	☐ Tendon ☐ Vessel/nerve ☐ Bone ☐ Joint ☐ Multiple
	12	Underlying fracture	☐ Yes ☐ No
	13	Surrounding skin	☐ Normal ☐ Hyperpigmented ☐ Hypopigmented ☐ Scar
C. Preoperative Wound Assessment	14	Wound discharge	☐ Absent ☐ Present: ☐ Blood ☐ Pus ☐ Serum
	15	Wound culture performed	☐ Yes ☐ No
		If positive, organism	__________________
		Antibiotic sensitivity	__________________
D. Operative Details	16	Date of operation	__________________
	17	Indication for reconstruction	__________________
	18	Defect size after excision/release	______ cm × ______ cm
	19	Flap size	______ cm × ______ cm
	20	Duration of operation	______ hours ______ minutes
	21	Donor-site closure	☐ Primary closure ☐ Split-thickness skin graft
	22	Early re-exploration for complication	☐ Yes ☐ No
		If yes, findings	__________________
		Management	__________________
E. Flap Survival and Complications	23	Flap viability	☐ Complete survival/no flap loss ☐ Marginal flap loss (<10%) ☐ Partial flap loss (10%-30%) ☐ Significant flap loss (>30%)
	24	Flap-related complications	☐ None ☐ Venous congestion ☐ Wound dehiscence ☐ Infection ☐ Other: __________
	25	Management of flap-related complication	☐ None ☐ Conservative ☐ Surgical intervention
F. Donor-Site Outcome	26	Donor-site complication	☐ None ☐ Wound dehiscence ☐ Graft loss
	27	Management of donor-site complication	☐ None ☐ Conservative ☐ Surgical intervention
G. Functional Outcome	28	Elbow range of motion at final follow-up	Flexion: ______° Extension: ______°
	29	Functional status	☐ Full range of motion ☐ Restricted movement ☐ No movement
H. Final Composite Reconstructive Outcome	30	Overall outcome	☐ Good ☐ Satisfactory ☐ Poor
		Good	Complete flap survival; no flap-related complications; no donor-site complications; full elbow range of motion.
		Satisfactory	Marginal or partial flap loss; conservatively managed flap-related or donor-site complications; and/or restricted elbow movement.
		Poor	Significant flap loss; flap-related or donor-site complications requiring surgical intervention; and/or no elbow movement.
